# Twitch user perceptions, attitudes and behaviours in relation to food and beverage marketing on Twitch compared with YouTube

**DOI:** 10.1017/jns.2021.22

**Published:** 2021-04-27

**Authors:** Catherine C. Pollack, Diane Gilbert-Diamond, Jennifer A. Emond, Alec Eschholz, Rebecca K. Evans, Emma J. Boyland, Travis D. Masterson

**Affiliations:** 1 Department of Biomedical Data Science, Geisel School of Medicine at Dartmouth College, Williamson Translational Research Building 3rd Floor, 1 Medical Center Drive, Lebanon, NH 03756, USA; 2 Department of Epidemiology, Geisel School of Medicine at Dartmouth College, Williamson Translational Research Building 7th Floor, 1 Medical Center Drive, Lebanon, NH 03756, USA; 3 Department of Pediatrics, Geisel School of Medicine at Dartmouth College, Dartmouth-Hitchcock Medical Center, 1 Medical Center Drive, Lebanon, NH 03756, USA; 4 Department of Psychology, University of Liverpool, Eleanor Rathbone Building, Bedford Street South, Liverpool L69 7ZA, UK; 5 Department of Nutritional Sciences, College of Health and Human Development, The Pennsylvania State University, 110 Chandlee Laboratory, University Park, PA 16802, USA

**Keywords:** Consumer behavior, Eating behavior, Food marketing, Livestreaming

## Abstract

Influencer marketing may be amplified on livestreaming platforms (e.g., Twitch) compared with asynchronous social media (e.g., YouTube). However, food and beverage marketing on Twitch has not been evaluated at a user level. The present study aimed to compare users’ self-reported exposure to food marketing and associated attitudes, consumption and purchasing behaviours on Twitch compared with YouTube. A survey administered via social media was completed by 621 Twitch users (90 % male, 64 % white, 69 % under 25 years old). Of respondents, 72 % recalled observing at least one food or beverage advertisement on Twitch. There were significant differences in the recall of specific brands advertised on Twitch (*P* < 0⋅01). After observing advertised products, 14 % reported craving the product and 8 % reported purchasing one. In chat rooms, 56 % observed conversations related to food and 25 % participated in such conversations. There were significant differences in the number of users who consumed various products while watching Twitch (*P* < 0⋅01). Of users who frequented YouTube (*n* 273), 65 % reported negative emotions when encountering advertising on YouTube compared with 40 % on Twitch (*P* < 0⋅01). A higher proportion felt Twitch's advertising primarily supported content creators (79 *v*. 54 %, *P* < 0⋅01), while a higher proportion felt that YouTube's advertising primarily supported the platform (49 *v*. 66 %, *P* < 0⋅01). The findings support that food marketing exposures on Twitch are noticeable, less bothersome to users and influence consumption and purchasing behaviours. Future studies are needed to examine how the livestreaming environment may enhance advertising effectiveness relative to asynchronous platforms.

## Introduction

Social media has transformed the traditional marketing space, bringing new opportunities and methods that companies can leverage to reach diverse populations. One of the most prominent new forms of advertising is influencer marketing, whereby popular online personalities collaborate with businesses to endorse and promote products to their users^(1)^. Influencer marketing is particularly noticeable among food and beverage products, and clear associations exist between influencer marketing of food and beverage products and various viewer behaviours, including brand recognition, desire to ‘like’ or ‘share’ posts and longitudinal consumption^(2,3)^. The impacts of influencer marketing may particularly be harmful to children and adolescents who may be unable to fully comprehend the misleading nature of the material^(4)^. Current policies in some countries (such as the United Kingdom) require influencers to disclose sponsorship relationships in their content so as to reduce deception^(5)^. Such disclosures have not been found to mitigate the effect of advertising exposure on consumption, and they may even increase the magnitude of the food intake response^(6)^. However, the majority of research focused on influencer marketing has been limited to social media platforms such as Facebook, Instagram and YouTube, where a majority of content is delivered asynchronously. Thus, there exists a dearth of research surrounding influencer marketing on social media platforms that specialize in livestreaming content.

‘Livestreaming’ is a content delivery method that broadcasts live, audiovisual material to international audiences. This form of entertainment is the cornerstone of the social media platform Twitch. Launched in 2011 and acquired by Amazon in 2014 for $970 million USD, Twitch is the leading provider of video game livestream material^(7,8)^. Over 4⋅5 billion hours of content were watched on Twitch in the third quarter of 2020, which also saw 2⋅15 million average concurrent viewers^(8)^. Twitch's primary audience of young adolescent and adult men is often considered one of the most elusive in advertising and fundamentally differs from other social media platforms (e.g., Instagram). This, coupled with the lack of scrutiny on advertising compared with other platforms, makes Twitch an ideal environment for companies to reach these new audiences^(9–12)^.

Beyond the expanded reach into a new demographic area, the livestreaming environment itself may enhance traditional influencer marketing by facilitating direct engagement between the streamer and the viewer. As the streamer broadcasts audiovisual content, users can interact with each other and the streamer through a real-time chat box placed near the broadcast. This is in stark contrast to asynchronous content sharing platforms such as Instagram or YouTube, whereby users primarily interact with the content creator through time-lagged comments, if at all. As a result, Twitch users possess strong feelings of connectivity with not only the influencer but also the larger community of viewers^(13)^. A recent study that found that a viewer's emotional connectedness to Twitch was significantly associated with an increased sense of community on the platform, which was also associated with a history of monetary donations to Twitch content creators^(14)^.

Despite the novelty of influencer marketing in a livestreaming environment, food and beverage mentions on Twitch are already prominent and pervasive. A recent evaluation of energy-dense, nutrient-poor food and beverage brands on Twitch found millions of exposure-hours in streamer profile panels, stream titles and chat room messages. This exposure had steadily increased over the 18-month evaluation period, suggesting that this type of advertising is growing on the platform^(15)^. Given the association between excessive intake of energy-dense, nutrient-poor foods and adverse health outcomes, it is imperative to evaluate viewers’ exposure to and perceptions of this type of marketing, as well as its corresponding impacts on consumption and purchasing behaviours^(16)^. Thus, the purposes of the present study were to (1) evaluate Twitch users’ experiences, attitudes and behaviours in relation to food and beverage marketing on Twitch and (2) directly compare and contrast these attitudes and behaviours to those associated with food and beverage marketing on an asynchronous social media platform (YouTube).

## Methods

### Survey design

A questionnaire was designed specifically for the present study to capture Twitch users’ engagement with and exposure to food and beverage product marketing on the platform, including perceptions towards advertising as well as consumption, craving and purchasing behaviours (the full questionnaire is available in the Supplementary Appendix of Supplementary material). Users were asked to recall whether they observed advertisements for nine product categories, including restaurants and food delivery services, snack foods, candies, sodas, energy drinks, coffees and teas and sports drinks. In addition, a list of twenty-nine brands were specifically asked about based on their high prevalence in Twitch streamer profile panels as determined by previous work^(15)^. The survey also asked participants about their behaviours and brand exposure on the user's two other most frequently used platforms (determined from self-report), as well as general demographic characteristics including gender, ethnicity, race and age. Inclusion criteria included an age above 13 (which was assessed throughout the survey via several ‘age checks’) and use of the Twitch platform (which was assessed in a short pre-screening questionnaire). Additionally, a series of focus questions were placed throughout the questionnaire to ensure participants were consciously considering their responses. The primary outcomes of interest were product exposure, product craving and purchasing, chat room exposure and engagement, product consumption and sentiment surrounding advertising. For the Twitch-specific analysis, the primary exposures of interest were monetary payment to support a streamer and daily viewership hours. For the Twitch-YouTube comparative analysis, the exposure of interest was the platform (Twitch *v*. YouTube). Specifics on each type of question are enumerated in the ‘Survey questions’ section.

The content aggregation platform Reddit was chosen as the primary advertising location for the survey given its similar demographic makeup to the Twitch platform of predominately young, college-aged men^(11,17)^. The survey was posted on twenty-three subreddits between 1 May and 23 May 2020. These subreddits included communities that were related to general video gaming (e.g., r/gamingsuggestions, r/MMORPG), those that were specific to the Twitch platform (e.g., r/Twitch, r/Twitch_Startup), and communities that were dedicated to one of the top 50 most frequently streamed games on Twitch (e.g., r/DotA2, r/skyrim)^(18)^. Surveys were created in and administered through the secure web application REDCap and were anonymous, but participants who reported their email were entered into a raffle for one of ten $50 USD Amazon Gift Cards^(19)^. The present study was conducted according to the guidelines laid down in the Declaration of Helsinki and all procedures involving human subjects were approved by the Trustees of Dartmouth College Committee for the Protection of Human Subjects (STUDY00032023). Written informed consent was obtained from all subjects.

### Survey questions

The following subsections specify the questions asked for each of the main variables that are reported in the ‘Results’ section. For all sets of questions, respondents were asked the same of their top two other most frequently used platforms.

### General product exposures

On a 4-point Likert scale ranging from ‘Never’ to ‘Always,’ users were asked to identify how often they saw advertising for the nine product categories while watching Twitch. They were also asked to check boxes for which of the specific twenty-nine products they had seen advertised on Twitch, including a free-response box to mention any other brands.

### Product craving and purchasing

Users were asked with a binary ‘yes/no’ question, ‘After seeing advertisements on Twitch do you crave any of the products that you see?’ If they selected ‘yes,’ users were then prompted to check one of the twenty-nine specific products they craved or specify an unlisted product in a free-response box. Users were also asked with a binary ‘yes/no’ question whether they ever purchased products because they had seen them advertised on Twitch. Users were again prompted to check one of the twenty-nine specific products or list any unlisted products.

### Chat room exposures and engagement

On a 4-point Likert scale ranging from ‘Never’ to ‘Always,’ users were asked how often they used the chatroom feature while watching Twitch; how often they saw other users talking about specific food and beverage products in the Twitch chatroom; and how often they themselves talked about specific food and beverage products in the Twitch chatroom.

### Product consumption

On a 4-point Likert scale ranging from ‘Never’ to ‘Always,’ users were asked how often they ate (or drank) one of the eight general product categories while watching Twitch. Users were also asked to rank how often they ordered food from meal delivery services (i.e., GrubHub and DoorDash) while watching Twitch.

### Sentiment surrounding Twitch advertising

Users were asked to fill in the blank: ‘On Twitch there is _____ advertising,’ with the options to select ‘Too little,’ ‘Just enough’ or ‘Too much.’ Users were also asked, ‘When I see advertising on Twitch, I am _____,’ with the option to select ‘Happy,’ ‘Annoyed’ or ‘Doesn't bother me.’ For three separate questions, a 4-point Likert scale (ranging from ‘Strongly Disagree’ to ‘Strongly Agree’) was used to asked users whether they thought the main purpose of advertising on Twitch was to ‘promote products,’ ‘support streamers/content creators’ or ‘increase profits for the website or app.’

### User stratification

In addition to standard, aggregate analysis, several sub-analyses were performed that stratified users into different groups based on their self-reported behaviours. In the first sub-analysis, participants were classified as either a ‘non-paying’ user of the Twitch platform or a ‘paying’ user. Here, ‘non-paying’ users were defined as individuals who had not spent any money on the Twitch platform. This included individuals who had neither purchased platform specific currency (Bits) nor subscribed to any channels, with the exception of individuals who had redeemed one free subscription through their Amazon Prime account. In contrast, users were classified as ‘paying’ if they had spent money on the platform through either a direct subscription to a channel or the purchase of Bits. One participant reported that they were a subscriber but were subscribed to zero channels, and this individual was subsequently removed from the analysis given their contradictory responses. This stratified analysis was motivated by variations in how channel subscribers view livestreams, as one of the major benefits to subscribing is the removal of pre-made video advertisements during the broadcast^(20)^.

In the second sub-analysis, participants were classified by their self-reported usage of the platform. Users were allocated into one of three categories: 0–2 h/d; 2–4 h/d; over 4 h/d. A third sub-analysis was performed to compare consumption behaviours, product craving and purchasing, observations of advertisements and sentiments towards advertisements between Twitch and the popular video sharing platform YouTube^(21)^. YouTube was selected as the comparison platform given its relative similarity of format to Twitch and its high utilization rate among participants.

### Statistical analysis

All Likert scales were collapsed into a bivariate response variable of either ‘Never’ (consisting of those who answered a question with ‘Never’) or ‘At Least Once’ (consisting of those who answered a question with ‘Rarely,’ ‘Sometimes,’ or ‘Often’) in order to simplify interpretation and minimize data interpretation errors that resulted from misclassification between the ‘Rarely’ and ‘Sometimes’ options (analysis with the full Likert scale is available in the Supplementary Appendix of Supplementary material). *χ*^2^ tests were performed for all categorical variable comparisons, including demographics comparisons; sentiments towards advertising on Twitch (including the frequency and purpose of advertisements); observations of product ‘mentions’ in the Twitch chat room (including comments by the user); the reported frequency of exposure to different product categories; and variations in the consumption of product categories. Each set of analyses was conducted three times: once in aggregate; once to compare answers between pay tiers; once to compare answers between viewership hours. Further, *χ*^2^ tests were performed to evaluate variations between specific product viewership, craving or purchasing by either product category, pay tier or viewership hours. Consumption and viewing behaviours were also compared across platforms (i.e., Twitch and YouTube) using *χ*^2^ tests. For all cases, a Fisher Exact test replaced a *χ*^2^ test when the expected frequencies for one of the groups were below five. In all cases where a *χ*^2^ test between pay tiers or viewership hours and the outcome of interest was significant, a subsequent multivariable logistic regression was run to adjust for demographic confounders. Before performing statistical analysis, a significance level of 0⋅05 was selected. All hypotheses surrounding the data were determined before the data collection process, as was the analytic plan. Analysis was conducted with the statistical software R (version 3.6.3, Vienna, Austria) in the RStudio graphical user interface (version 1.3.959, Boston, MA, USA).

### Sensitivity analyses

Sensitivity analyses were conducted across all measures with the full Likert scale in order to ensure that no underlying relationships were masked by collapsing the Likert scale^(22)^. The results were similar between the collapsed and the original scale, and the collapsed results are presented for simplified interpretation (see Supplementary Appendix of Supplementary material for results with the complete Likert scale). In a separate sensitivity analysis, users who had not purchased Bits but had subscribed to a channel through their Amazon Prime account were separated from users who had neither bought Bits nor subscribed to any channel. However, these results did not dramatically differ from those of the binary classification and were, thus, collapsed for simplicity (see Supplementary Appendix of Supplementary material).

## Results

### Participants

**Fig. 1.**
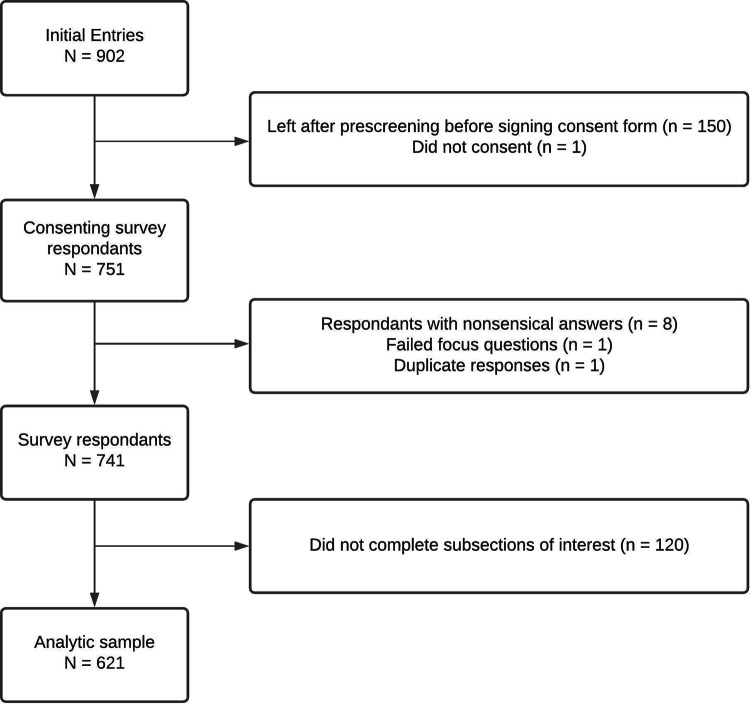
Participant flow diagram.

There were 902 individuals who completed the initial pre-screening for the survey, with 751 advancing to the actual survey. Of these participants, 120 were removed due to missing data, while 9 were removed for either failing an age or focus check or other miscellaneous preprocessing reasons (including a screening for duplicates). This resulted in 671 participants who were included in the analytic data set (Fig. 1).

Participants were predominately male (90 %) and of non-Hispanic or Latino ethnicity (80 %). The majority of participants were White (64 %), followed by Asian (24 %). A plurality of respondents were between 18 and 24 years old (44 %), followed by 25–34 (27 %) and under 18 (26 %). The age, ethnicity and gender distributions were similar to previously reported statistics on Twitch's viewership audience that were last released in 2017^(9)^. There were no significant differences in age, gender or ethnicity between those who completed the questionnaire (*n* 621) and those who did not (*n* 170; Table 1). A small but significant difference in reported race was observed between the full and the reduced sample, with a slightly higher percentage of Asian individuals in those who completed the questionnaire (23⋅3 % compared with 17⋅6 %). Taken together, this lack of significance demonstrates that non-response bias due to demographic features was negligible.

**Table 1.** Demographic characteristics

Characteristic *N* (%)	Complete data set *N* 620	Missing data *N* 170	*P*-value*
Gender			0⋅2
Male	563 (90⋅2 %)	111 (65⋅3 %)	
Female	53 (8⋅5 %)	20 (11⋅8 %)	
Other	5 (0⋅8 %)	1 (0⋅6 %)	
Did Not Answer	–	38 (22⋅4 %)	
Ethnicity			0⋅2
Not Hispanic or Latino	501 (80⋅7 %)	108 (63⋅5 %)	
Hispanic or Latino	80 (12⋅9 %)	10 (5⋅9 %)	
Unknown	11 (1⋅8 %)	2 (1⋅2 %)	
Prefer Not to Answer	29 (4⋅7 %)	11 (6⋅5 %)	
Did Not Answer	–	39 (22⋅9 %)	
Race			< 0⋅01
White	398 (64⋅1 %)	84 (49⋅4 %)	
American Indian or Alaska Native	11 (1⋅8 %)	3 (1⋅8 %)	
Asian	145 (23⋅3 %)	30 (17⋅6 %)	
Black or African American	23 (3⋅7 %)	2 (1⋅2 %)	
Native Hawaiian or Pacific Islander	2 (0⋅3 %)	–	
Other	38 (6⋅1 %)	10 (5⋅9 %)	
Unknown	7 (1⋅1 %)	1 (0⋅6 %)	
Prefer Not to Answer	33 (5⋅3 %)	11 (6⋅5 %)	
Did Not Answer	–	29 (17 %)	
Age			0⋅5
Under 18	161 (25⋅9 %)	28 (16⋅5 %)	
18–24	269 (43⋅3 %)	63 (37⋅1 %)	
25–34	168 (27⋅1 %)	35 (20⋅6 %)	
35–44	20 (3⋅2 %)	3 (1⋅8 %)	
45 and Above	3 (0⋅5 %)	2 (1⋅2 %)	
Did Not Answer		39 (22⋅9 %)	

* All *P*-values are from a *χ*^2^ test. Note that the ‘Did Not Answer’ category was not included in the tests.

### General product exposure

**Fig. 2.**
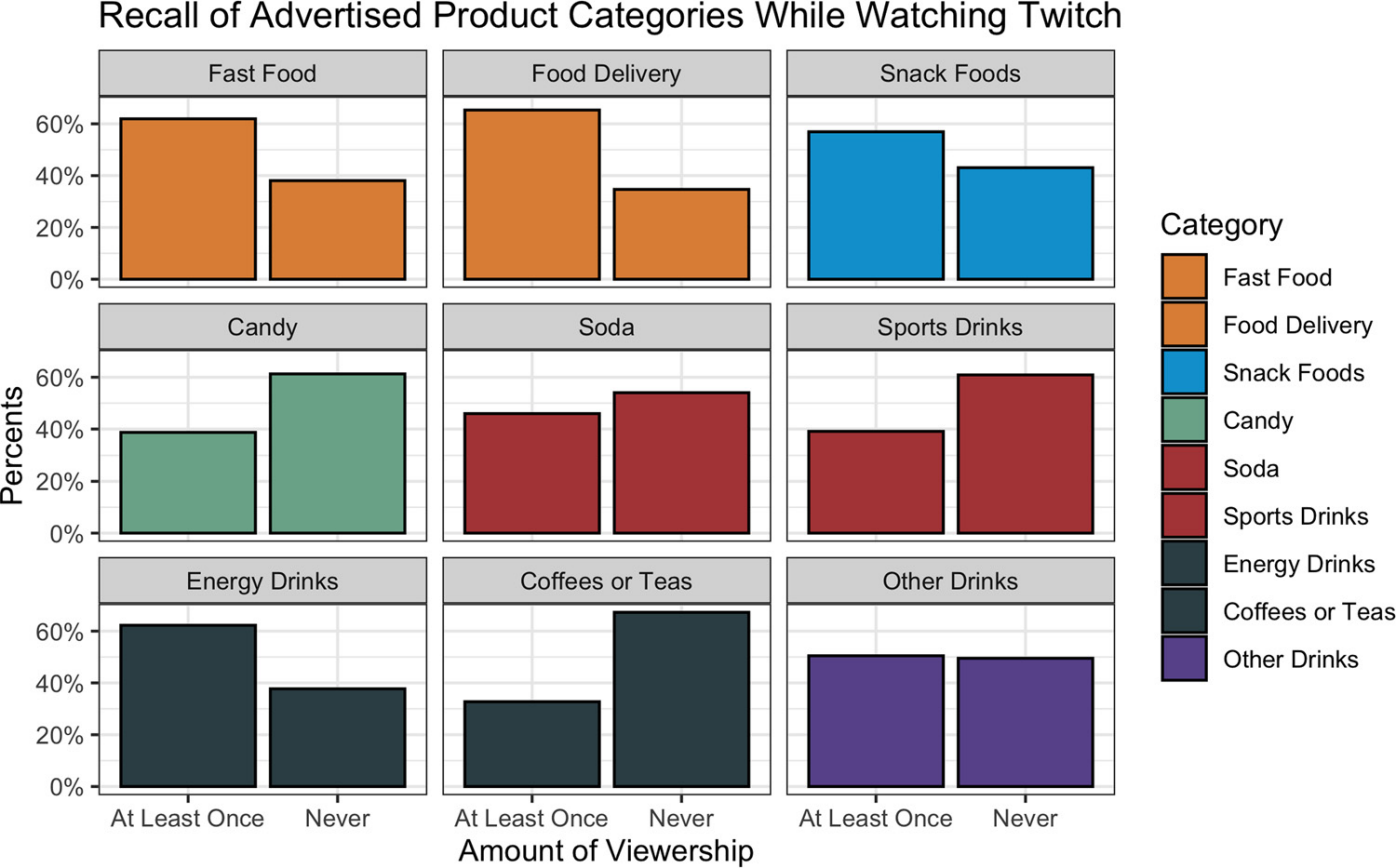
Recall of advertised products while watching Twitch by product category. Amount of viewership determined by a collapsed 4-point Likert scale ranging from ‘never’ to ‘often,’ with ‘rarely’, ‘sometimes’ and ‘often’ collapsed into ‘at least once.’

There was a significant difference in recalled advertising to food and beverage products on Twitch between product categories (*P* < 0⋅01; Fig. 2). Of the nine product categories asked about in the survey, there were five in which a majority of users reported seeing at least one advertisement on Twitch: food delivery services (65 %); fast-food restaurants (62 %); energy drinks (62 %); snack foods (57 %); ‘other drinks’ (50 %). There were no significant differences between either the pay tiers or viewership hours and the frequency of recalled advertisements while watching Twitch for any of the product categories.

**Fig. 3.**
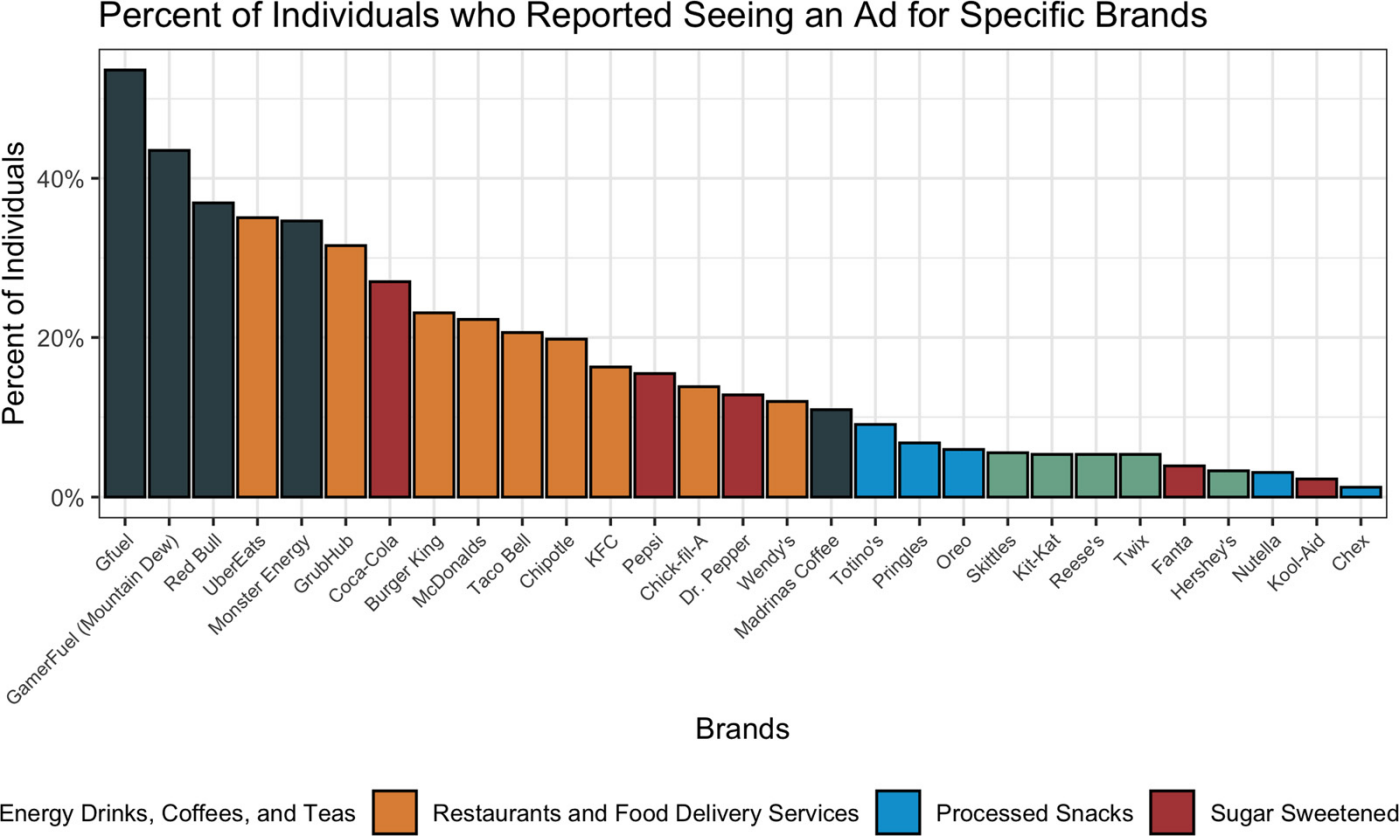
User-reported brand-specific advertisements on Twitch. Percentages are out of those who reported seeing at least one of the twenty-nine mentioned brands (*n* 485).

When asked about exposures to specific brands, 78⋅1 % (*n* 485) of users recalled seeing an advertisement on Twitch for at least one of the brands included in the list (Fig. 3). Generally, more users reported viewing specific energy drink, coffee or tea brands than any other category, followed by restaurants and food delivery services, sugar sweetened beverages, processed snacks and candy brands. Of those who reported seeing a brand, the most commonly recalled were Gfuel (54 %), followed by GamerFuel/Mountain Dew (44 %), Red Bull (37 %), UberEats (35 %) and Monster Energy (35 %).

### Product craving and purchasing

**Fig. 4.**
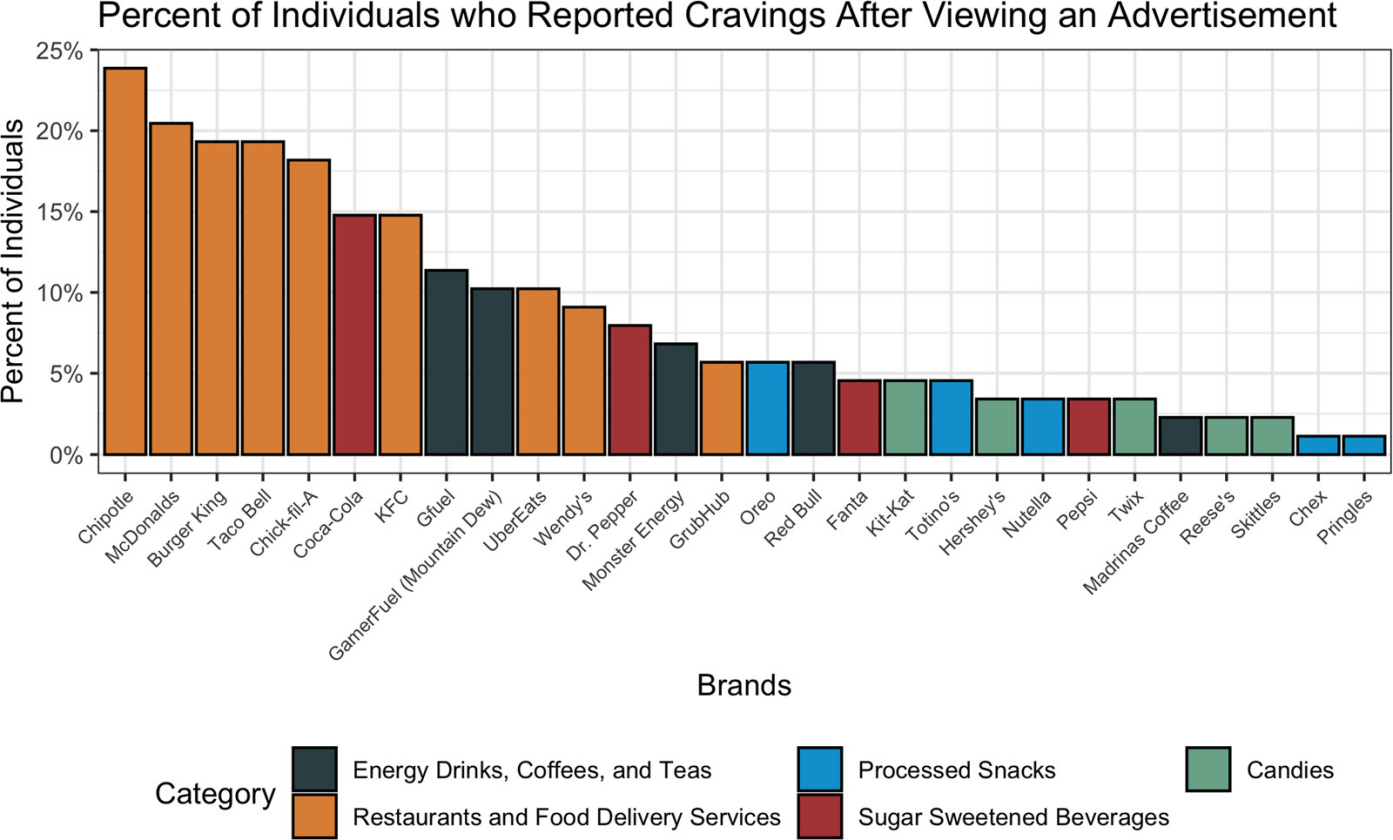
Distribution of product-specific cravings after viewing the advertisement on Twitch. Percentages are out of those who reported craving at least one of the twenty-nine mentioned brands (*n* 88).

A total of 14 % of individuals in the survey (*n* 88) recalled craving a product after they had seen it advertised on Twitch. When asked about specific brands, eight of the top ten observed brands were for fast food or food delivery (Chipotle, McDonalds, Burger King, Taco Bell, Chick-fil-A, KFC, UberEats and Wendy's; Fig. 4). There was no significant difference across viewership hour groups and reported craving of a brand. However, there was a significant difference between pay tiers (*P* = 0⋅02). Specifically, 19 % (*n* 43) of paying users reported craving a product, compared with 12 % (*n* 45) of non-paying users. This effect remained significant after adjusting for demographic covariates (OR 1⋅07, 95 % CI 1⋅01, 1⋅14, *P* = 0⋅01).

**Fig. 5.**
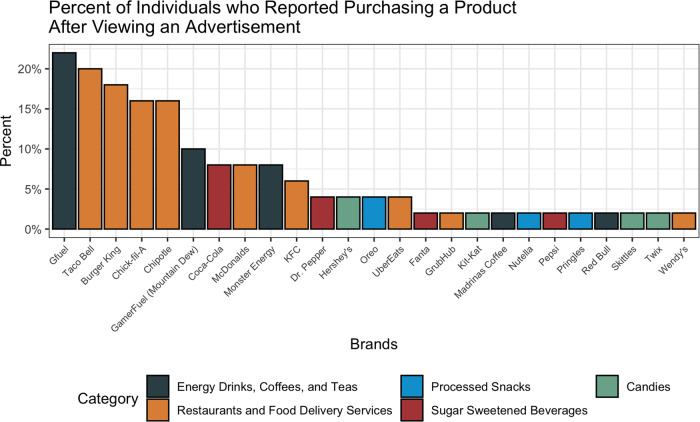
Distribution of product-specific purchasing after viewing the advertisement on Twitch. Percentages are out of those who reported seeing at least one of the twenty-nine mentioned brands (*n* 50).

Of the survey respondents, 8 % (*n* 50) reported purchasing a product related to a brand after they had seen it advertised on Twitch (Fig. 5). The most purchased products included Gfuel (22 %, *n* 11), Taco Bell (20 %, *n* 9), Burger King (18 %, *n* 9), Chick-Fil-A (16 %, *n* 8) and Chipotle (16 %, *n* 8). There was a significant difference between the pay tiers in the proportion of individuals who purchased a product after they saw it advertised (*P* < 0⋅01). Specifically, 13 % of paying users (*n* 29) reported purchasing a product after viewing the advertisement compared with just 5 % (*n* 21) of non-paying users. This effect remained significant after adjusting for demographic covariates (OR 1⋅07, 95 % CI 1⋅02, 1⋅12, *P* < 0⋅01). There were also significant differences between the number of hours of viewership on Twitch and whether an individual purchased a product after seeing it advertised (*P* < 0⋅01). In particular, 12 % of users who spent 2–4 h (*n* 16) or over 4 h (*n* 10) on Twitch reported purchasing advertised products compared with 6 % (*n* 24) of under-2 h users. After adjusting for demographic covariates, there was still a significant difference between the under 2 h and the 2–4 h categories (OR 1⋅06, 95 % CI 1⋅01, 1⋅12, *P* = 0⋅03), but there was no significant difference between the under 2 h and the over 4 h categories.

### Chat room exposures and engagement

The majority of users (56 %, *n* 347) reported observing food and beverages discussed in the Twitch chat room at least once, with 25 % (*n* 158) noting that they had actively engaged in these conversations. There was a significant difference in the frequency of conversations observed by pay tier (*P* < 0⋅01). Specifically, 63 % (*n* 147) of paying users reported observing conversations about products in the Twitch chat rooms, compared with 52 % (*n* 200) of non-paying users. This association remained significant after adjusting for sociodemographic covariates (OR 1⋅14, 95 % CI 1⋅05, 1⋅24, *P* < 0⋅01). There was also a significant difference (*P* < 0⋅01) in whether a user had engaged in conversations on food and beverage products, with 31 % (*n* 74) of paying users reporting involvement compared with 22 % (*n* 84) of non-paying users. This also remained significant after adjusting for sociodemographic covariates (OR 1⋅10, 95 % CI 1⋅02, 1⋅18, *P* = 0⋅01).

Furthermore, there were significant differences (*P* < 0⋅01) between viewership categories and whether a user had reported observing conversations on food and beverage products in the Twitch chat room. Of note, 74 % (*n* 61) of users who used Twitch over 4 h/d acknowledged that they had seen product conversations at least once compared with 58 % (*n* 76) of users who spent 2–4 h/d on the platform and 51 % (*n* 210) of users who spent under 2 h on the platform. However, only the difference between under 2 h users and over 4 h users remained significant after adjusting for sociodemographic covariates (OR 1⋅27, 95 % CI 1⋅12, 1⋅43, *P* < 0⋅01). There were also significant differences (*P* < 0⋅01) in whether the user had themselves discussed the products, with 44 % (*n* 36) of ‘over 4 h’ users, 29 % (*n* 38) of ‘2–4 h’ users, and 21 % (*n* 84) of ‘under 2 h’ users participating in conversations related to food and beverage products. After adjusting for sociodemographic covariates, only the difference between under 2 h viewers and over 4 h viewers was significant (OR 1⋅23, 95 % CI 1⋅11, 1⋅37, *P* < 0⋅01).

### Product consumption

**Fig. 6.**
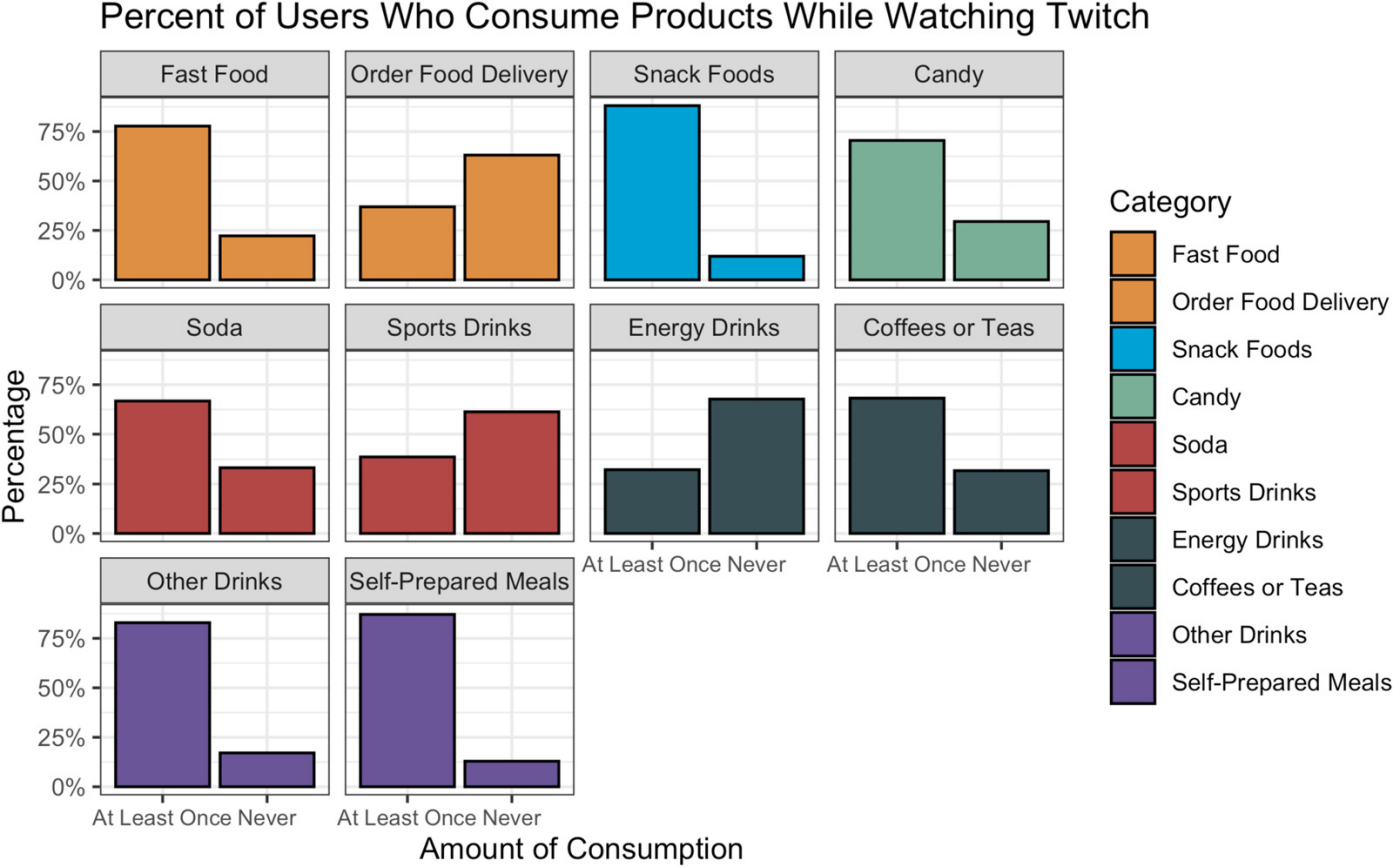
Comparison of consumption behaviours while watching Twitch across product categories. Amount of viewership determined by a collapsed 4-point Likert scale ranging from ‘never’ to ‘often,’ with ‘rarely,’ ‘sometimes’ and ‘often’ collapsed into ‘at least once.’

There were significant differences (*P* < 0⋅01) in the reported consumption of the various product categories by users while watching Twitch (Fig. 6). Six of the eight product categories were reportedly consumed by a majority of respondents at least once while viewing Twitch. The most frequently reported were snack foods (88 %), ‘other drinks’ (83 %) and fast food (78 %). Additionally, 87 % of respondents reported consuming self-prepared meals while watching Twitch, with only 37 % ordering food delivery while watching Twitch. However, a significantly larger proportion of users who paid for Twitch reported ordering food delivery (44 %, *n* 103) compared with non-paying users (32 %, *n* 126, *P* < 0⋅01). This remained significant when adjusting for sociodemographic covariates (OR 1⋅09, 95 % CI 1⋅01, 1⋅18, *P* = 0⋅03). This significant variation was also present when stratifying by viewership categories in both the unadjusted (*P* < 0⋅01) and the adjusted (2–4 h OR 1⋅13, 95 % CI 1⋅03, 1⋅24, *P* = 0⋅01; over 4 h OR 1⋅17, 95 % CI 1⋅04, 1⋅31, *P* < 0⋅01) models. A majority of individuals who spent over 4 h on Twitch had ordered meal delivery while viewing Twitch (51 %, *n* 42) compared with 46 % (*n* 60) of those who spent 2–4 h/d on the platform and 31 % (*n* 127) of those who spent under 2 h/d on the platform. In addition, a higher proportion of users who viewed Twitch over 4 h/d reported consumption of snack foods (96 %, *n* 79, *P* = 0⋅03) while watching Twitch compared with the 2–4 h group (89 %, *n* 116) and the under 2 h group (86 %, *n* 351). However, after adjusting for sociodemographic covariates, only the over 4 h group was statistically different from the under 2 h group (OR 1⋅13, 95 % CI 1⋅05, 1⋅22, *P* < 0⋅01). All other product categories were not significantly different between paying and non-paying users or between viewership categories.

### Sentiments surrounding Twitch advertising

No significant differences were found between a user's pay tier or level of viewership and their level of agreement on whether the main purpose of advertising on Twitch was to promote products, support streamers and content creators or increase profits for the website. Further, no significant difference was found between the amount of perceived advertising on Twitch and the user's pay tier or viewership hours. In addition, no significant difference was found between the user's pay tier or viewership hours and how advertisements on Twitch made them feel.

### Cross-platform behaviours

**Fig. 7.**
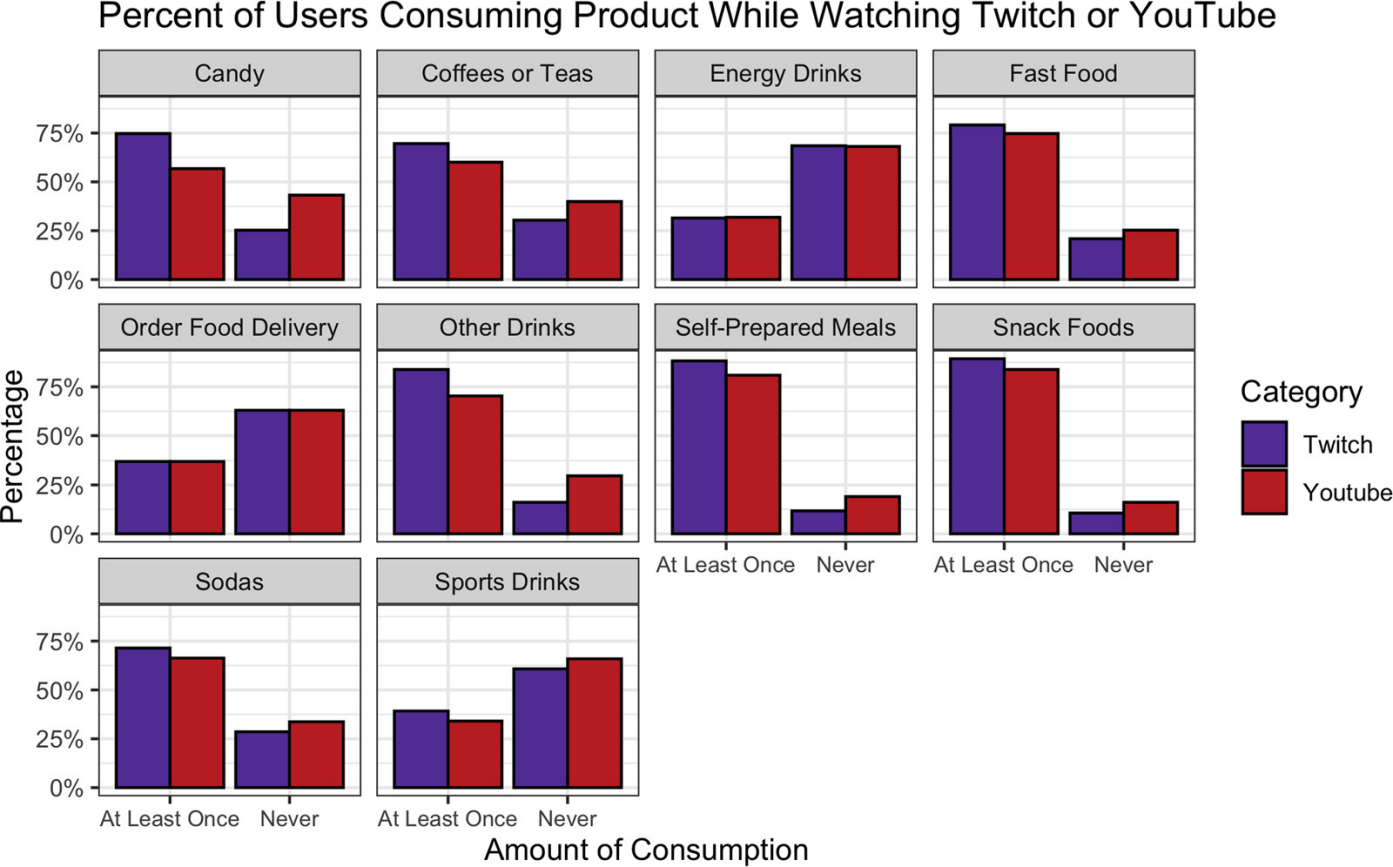
Comparison of cross-platform consumption behaviours across product categories. Amount of viewership determined by a collapsed 4-point Likert scale ranging from ‘never’ to ‘often,’ with ‘rarely,’ ‘sometimes’ and ‘often’ collapsed into ‘at least once.’

There were 273 users who reported YouTube as one of their top two most frequently used platforms and completed the corresponding consumption and behavioural questions about the platform. Fig. 7 compares the consumption behaviours between this subset of users when they watch Twitch compared with when they watch YouTube. Three of the eight product categories were significantly different between Twitch and YouTube (*P* < 0⋅01). Significantly more users reported consuming candy (75 %, *n* 204), coffee or teas (70 %, *n* 190) and ‘other drinks’ (84 %, *n* 229) while watching Twitch compared with when they watched YouTube (57, 60 and 70 %, respectively). Additionally, more users (88 %, *n* 241) reported consuming a self-prepared meal at least once while they watched Twitch compared with when they watched YouTube (81 %, *n* 221). All other product categories were not significantly different between Twitch and YouTube.

Table 2 compares other food marketing-related behaviours of users between Twitch and YouTube. There were no significant differences in product cravings or purchasing between the platforms. Yet, there were significant differences in the engagement of users with products. Significantly more individuals (*P* < 0⋅01) reported observing fellow users discuss food and beverage products on Twitch (53 %, *n* 145) compared with YouTube (33 %, *n* 91). Individuals also reported more engagement in product-related conversations on Twitch (25 %, *n* 67) compared with YouTube (13 %, *n* 35, *P* < 0⋅01). There were also variations in users’ perceptions towards general marketing practices between Twitch and YouTube. For example, while 60 % (*n* 135) of respondents felt there was ‘too much’ advertising on YouTube, 68 % (*n* 187) of the same group of users reported there was ‘just enough’ advertising on Twitch *P* < 0⋅01). There were also differences in how advertisements on the platform made the users feel. A majority of the sample (65 %, *n* 178) were ‘annoyed’ when they saw advertisements on YouTube compared with only 40 % on Twitch (*n* 109, *P* < 0⋅01); in fact, the majority of users reported that they were not bothered by the presence of advertisements on Twitch (59 %, *n* 162). Furthermore, there were significant variations in the identified purpose of advertisements between platforms. For Twitch, 79 % of users (*n* 215) agreed or strongly agreed that advertisements primarily served to support streamers and content creators. However, only 54 % of users (*n* 149) thought this was true on YouTube (*P* < 0⋅01). In contrast, 66 % of users ‘strongly agreed’ that the purpose of advertisements on YouTube was to increase YouTube's profits as a company, compared with just 49 % of users who felt the same was true of Twitch (*P* < 0⋅01). Similarly, 44 % of users ‘strongly agreed’ that the purpose of advertisements on YouTube was to promote products, compared with 33 % of users who felt this was true of Twitch (*P* = 0⋅04).

**Table 2.** Cross-platform behaviours

Behaviour	YouTube *N* 273	Twitch *N* 273	*P*-value*
Crave Product After Viewing Advertisement			0⋅72
No	228 (84 %)	232 (85 %)	
Yes	45 (16 %)	41 (15 %)	
Purchase Product After Viewing Advertisement			0⋅23
No	244 (89 %)	253 (93 %)	
Yes	29 (11 %)	20 (7 %)	
Observe Others Discussing Products			*P* < 0⋅01
Never	182 (67 %)	128 (47 %)	
At Least Once	91 (33 %)	145 (53 %)	
User Discusses Products			*P* < 0⋅01
Never	238 (87 %)	206 (75 %)	
At Least Once	35 (13 %)	67 (25 %)	
Amount of Advertising			*P* < 0⋅01
Too Little	5 (2 %)	10 (4 %)	
Just Enough	103 (38 %)	187 (68 %)	
Too Much	165 (60 %)	76 (28 %)	
Feeling When Seeing Advertisements			*P* < 0⋅01
Happy	2 (1 %)	2 (1 %)	
Annoyed	178 (65 %)	109 (40 %)	
Unbothered	93 (34 %)	162 (59 %)	
Advertisement Purpose to Promote Products			0⋅04
Strongly Agree	120 (44 %)	91 (33 %)	
Agree	118 (43 %)	128 (47 %)	
Disagree	25 (9 %)	39 (14 %)	
Strongly Disagree	10 (4 %)	15 (5 %)	
Advertisement Purpose to Support Streamers/Content Creators			*P* < 0⋅01
Strongly Agree	55 (20 %)	81 (30 %)	
Agree	94 (34 %)	134 (49 %)	
Disagree	81 (30 %)	44 (16 %)	
Strongly Disagree	43 (16 %)	14 (5 %)	
Advertisement Purpose to Increase Website's Profit			*P* < 0⋅01
Strongly Agree	180 (66 %)	134 (49 %)	
Agree	78 (29 %)	119 (45 %)	
Disagree	13 (5 %)	16 (6 %)	
Strongly Disagree	2 (1 %)	4 (1 %)	

* All *P*-values are from a *χ*^2^ test.

## Discussion

Food and beverage marketing on Twitch was noticed by a substantial proportion of the survey sample. The frequency in which individuals reported certain product categories is consistent with prior literature. Specifically, energy drinks were noticed by the highest proportion of participants, followed by restaurants and food delivery services, sodas, processed snacks and candies^(15)^. Interestingly, the lack of a significant difference between paying and non-paying users in terms of noticed advertisements suggests that purchasing a subscriptions to a channel does not fully protect a user from encountering product mentions despite the fact that pre- and mid-roll advertisements are removed from subscribed channels^(23)^. This emphasizes the variety of ways in which products can be promoted on Twitch beyond the traditional video advertisement, including turning the stream itself into an advertisement through paid segments, competitions and channel mini-games^(24)^. Additionally, the present survey showed no significant differences in the recall of advertised brands between the different viewership categories. This may suggest that food and beverage advertising is so pervasive on Twitch that it is consistently encountered, even if a user only briefly engages with the platform.

Despite the fact that energy drinks were the most noticed brands advertised on Twitch, the most craved brands were fast-food restaurant brands such as Chipotle, McDonalds and Burger King. This pattern was also seen for product purchases, although only 8 % of survey respondents actually reported purchasing a product following exposure to the advertisement. While the percentage of users who recalled purchasing a product following advertisement exposure may seem small, Twitch averages 17⋅5 million average daily visitors; this suggests that a sizable number of individuals (1⋅4 million, or 8 % of average daily visitors) may still be directly influenced to make purchases of various food and beverage products they see advertised on the platform, assuming that this sample is representative of Twitch's user base^(25)^. Further, marketing campaigns do not necessarily have to be tied to purchases as a metric for success, as repeat exposures can still influence brand loyalty and impact behaviours like consumption over a longer term^(26,27)^. Importantly, the proportion of users who reportedly purchased products following advertisements was observed to be significantly greater among those who had paid money to Twitch, but there was no significant difference with regard to the viewership hours. This may be suggestive of successful influencer marketing campaigns, as individuals who pay to support a streamer may also opt to purchase products as an indirect way of showing support regardless of the amount of time they engage with the stream. This is supported by prior literature in children that demonstrates how a viewer can establish loyalty towards a favourite online influencer and support them entirely, including their decision to promote products^(28)^.

The high engagement surrounding brands in the chat room is also of note. During extremely popular livestreaming events, the chat room may be limited to paid subscribers in order to preserve some level of engagement between the streamer and dedicated viewers. The fact that a significantly higher proportion of paid users actively discussed food and beverage products compared with non-paying users may be a result of brand-specific conversations during livestream marketing campaigns that have a subscriber-limited chat room. Significant differences in chat room engagement around food products were also found between viewership categories, which may suggest that users who engage with Twitch more frequently have more opportunities to engage with a sponsored livestreaming event. Indeed, chat rooms are frequently leveraged during marketing campaigns to enhance discussion about the target product; in these situations, chat room moderators may choose to remove negative posts related to the brand or, in one documented case, a user-generated comment that asked why the brand of interest was ‘contributing to childhood obesity’^(29)^. Given that chat room engagement has been associated with higher levels of satisfaction with the platform, high levels of engagement with the chat room, coupled with a brand's ability to direct the conversation surrounding the product of interest, may further compel individuals who actively engage with the platform to purchase or consume the sponsored products^(13)^.

Almost all food and beverage categories were reported to be consumed by a majority of respondents while actively watching Twitch. However, a higher proportion of paying users reported ordering food by meal delivery services while watching Twitch compared with non-paying users. A higher proportion of users who watched Twitch over 4 h/d also ordered meal delivery more than those in lower viewership categories, especially those who watched Twitch for under 2 h/d. Interestingly, some of the most prolific Twitch streamers and eSports teams are directly sponsored by major meal delivery services, emphasizing the power of influencer marketing despite the potential removal of video advertisements^(30,31)^. In addition to food delivery services, a larger proportion of users who spent over 4 h/d on Twitch reported the consumption of snack foods while watching Twitch compared with other viewership categories. These general trends in consumption behaviour in this primarily adult sample differ from a recent systematic review and meta-analysis of the association between advertising and consumption, which found a significant association for children but not adults^(32)^. Given that previous studies in adults have primarily focused on traditional advertising platforms such as television, the results of the present study demonstrate the potential power that direct engagement with influencers has on consumption and purchasing behaviours for adolescents and young adults. Therefore, future studies are needed to more fully understand the direct effects of acute and chronic exposure to influencer marketing on this demographic.

Influencer marketing is not constrained to Twitch, and it is often implemented on asynchronous social media platforms such as YouTube or Instagram. Studies evaluating food marketing on these platforms have shown that energy-dense, nutrient-poor foods are also prevalent on these platforms, and viewers actually tend to consume more of the advertised products when advertising is disclosed^(6,33)^. Despite YouTube's sensational popularity (73 % of US adults use YouTube), its focus on asynchronous content delivery, coupled with the absence of a live chat room, naturally creates a barrier between the influencer and the viewers^(34,35)^. This lack of direct engagement may be responsible for the stark differences in users’ perceptions on the purpose on advertising on both platforms. Given that users felt that Twitch's primary purpose for advertising was to support content creators (as opposed to YouTube, where users felt the primary purpose was to generate revenue for the platform), the same individuals who are dissuaded by YouTube food and beverage marketing could become loyal to the same brand if they saw it instead advertised by a favourite Twitch streamer. Additionally, other platforms such as YouTube Live or Facebook Gaming are beginning to promote livestreaming content delivery; therefore, this type of advertising may become more pervasive over time^(8)^.

### Limitations and future directions

Recruitment for respondents only occurred on the social media platform Reddit, and advertisements for the survey were specifically targeted towards Twitch users. While this guaranteed that Twitch users were reached effectively, it may have excluded individuals who use Twitch but not Reddit. However, Reddit and Twitch are fundamentally similar in their target user demographic, and the demographics of survey respondents mimic those reported by Twitch^(11)^. A second limitation in the present study is the subjective nature of the Likert scale options. This may have resulted in some misinterpretation by those taking the survey; however, this was mitigated by combining the ‘Rarely,’ ‘Sometimes’ and ‘Often’ categories into an ‘At Least Once’ category. Other limitations include the self-report nature of the survey, which relied on participants’ recall of advertisements and may have resulted in an underestimation of the true amount of food and beverage advertising on the platform. Further, the cross-sectional nature of the survey precludes the ability to establish a causal association between recalled advertisements and longitudinal craving or purchasing behaviours. Lastly, the survey instrument used here was generated specifically for the present study and has yet to be formally validated.

Future analysis could evaluate how engagement and consumption behaviours on Twitch compare with other platforms, including those with a similarly prominent influencer atmosphere (such as Instagram), those that also are known for livestreaming videogames (such as Facebook Gaming) or those where individuals may directly interact with the brand itself as opposed to an influencer (such as Twitter). Future work could also ask users for more specific quantitative estimates for certain questions to receive more precise responses, like asking users to state how many times per week they simultaneously watch Twitch and order food delivery. Additionally, given the high consumption of ‘other drinks’ reported while watching Twitch, future work could more precisely examine users’ use of and engagement with alcohol or tobacco brands while watching Twitch. Furthermore, given that the data collection was conducted during the novel coronavirus disease 2019 global pandemic, future studies should compare these findings against post-pandemic behaviours.

## Conclusions

The present study is the first to report on Twitch users’ perceptions, engagement and consumption behaviours related to food and beverage marketing on the platform. Significant recall differences were found between product categories, with fast-food and food delivery services, energy drinks and snack food all reportedly viewed by a majority of users. The present results suggest that the community fostered by the Twitch livestreaming environment may have a meaningful and major impact on a user's food and beverage consumption and purchasing behaviours. Future policy surrounding digital food and beverage marketing should consider how the livestreaming environment may be specifically regulated to limit children and adolescents’ exposure to energy-dense, nutrient-poor products.

## Supplementary material

For supplementary material accompanying this paper visit http://dx.doi.org/10.1017/jns.2021.22. click here to view supplementary material
