# Supplementary material for: Twitch user perceptions, attitudes and behaviours in relation to food and beverage marketing on Twitch compared with YouTube
Source: J Nutr Sci. 2021 Apr 27;10:e32. doi: 10.1017/jns.2021.22 (PMC8141682; doi:10.1017/jns.2021.22)
Supplement: Supplementary file 1 [file jnssup.zip › S2048679021000227sup002.pdf]

Dartmouth Media and Health Behaviors Lab

If you have questions at any point point please contact media.and.health@dartmouth.edu

Thank you for your interest in this online research survey! Please answer these pre-survey questions now before continuing!

Do you use Twitch?

◯ Yes
◯ No

Do you have a cell phone or smart phone?

◯ Yes
◯ No

Do you own a computer?

◯ Yes
◯ No

Do you primarily use a desktop or a laptop?

◯ desktop
◯ laptop

Do you wear glasses?

◯ Yes
◯ No

Do you have a pet dog or cat?

◯ Yes
◯ No

How old are you?

____

Unfortunately you are not eligible to take this survey. Thank you for your time!

◯ Click here to close this page

Welcome!
Thank you for your interest in this online research survey! Your participation would require taking a survey that may take 15 minutes to complete. The survey may include some questions about your attitudes towards marketing across social media platforms. Once you finish, you will have the option of entering a raffle to win one of 10 Amazon gift cards worth $50. To enter the raffle, you will need to enter your email address. Your email is only used to enter into the raffle and as a contact if you win and will be kept completely separate from your responses to all survey questions.

To complete the consent form please read through the form. If you wish to participate, check the box that says "I agree" and click next page. You will be asked to review your submission, and once you hit submit then you can begin the survey.

CONSENT TO TAKE PART IN RESEARCH

Dartmouth College
Media and Health Behaviors Lab at Dartmouth College
Media.and.Health@Dartmouth.edu

You are being asked to participate in a research study. Taking part in research is voluntary. You may choose to stop taking part in this study at any time with no consequences. You may choose to not answer any or all questions.

What is this study's purpose? We are studying attitudes towards a variety of marketing techniques across social media platforms.

Will you benefit from taking part in this study? You will likely not personally benefit from being in this study. We hope our research may help people in the future.

What does this study involve? Participating in this study will require up to 15 minutes to complete an online survey.

What is the risk involved with being enrolled in this study? There is very little risk involved in this study. Your privacy will be protected. Members of our study team will only use data for research purposes.

How will your privacy be protected? We will collect information about your social media use and your attitudes, beliefs and behaviors about foods and drinks by survey. We will not be going into your social media accounts to collect data. All of the information you provide will be kept anonymous and confidential. Your data will only be identified by a random study identification number. If you want to be entered into the raffle to win an Amazon.com gift card, you will need to provide a valid email address. That email address will not be linked to your survey responses, and we will delete your email address after completion of the raffle.

All data we collect will be saved as an encrypted database on a secure Dartmouth server and will be password protected. When the data are no longer needed, the data will be permanently deleted from the database. The information collected for this study will only be used for the research purposes stated earlier in this form.

Will you be paid to take part in this study? No. You may enter a raffle to win one of ten $50 e-gift cards to Amazon.com. If you want to enter that raffle, we will ask for your email address at the end of the survey. We will only use that email address to notify you of the raffle results. Your email address will not be linked to the study data. You will be notified of the raffle results via e-mail by July 31st, 2020.

Whom should you call with questions about this study? If you have questions or concerns about this study, you can contact the study team at Media & Health Behaviors Lab media.and.health@dartmouth.edu.

v. 5/13/2020
CPHS # 32023

CONSENT

Please read the following statement. If you agree, you will begin the study. Please print a copy of this form for your records before submitting.

I have read the above information and I agree to take part in this study.

☐ I agree and confirm I am 13 years of age or older.

☐ I do not want to participate in this study, or I am under the age of 13 years.

CONSENT
Please read the following statement. If you agree, you will begin the survey. Please print a copy of this form for your records before submitting.

I have read the above information and I agree to take part in this study.

◯ I agree
◯ I do not agree and I do not want to participate in the study

In what year were you born?

____

To which gender identity do you most identify?

- ◯ Female
- ◯ Male
- ◯ Other

If other please specify:

____

What is your ethnicity?

- ◯ Hispanic or Latino
- ◯ Not Hispanic or Latino
- ◯ Unknown
- ◯ I prefer not to answer

What is your race? Please select all that apply:

- ☐ American Indian or Alaskan
- ☐ Asian
- ☐ Black or African American
- ☐ Native Hawaiian or other Pacific Islander
- ☐ White
- ☐ Unknown
- ☐ Other
- ☐ I prefer not to answer

If other, please specify:

____

Are you currently a student?

- ◯ Yes
- ◯ No

If you are a student what grade are you currently in?

- ◯ Grade 8 or less
- ◯ High school
- ◯ College
- ◯ Professional or Graduate School

What is the highest level of education that you have completed?

- ◯ Grade 8 or less
- ◯ Some high school
- ◯ High school graduate or GED
- ◯ Some post-high-school, no degree
- ◯ Associates degree
- ◯ Bachelors degree
- ◯ Professional school or graduate school
- ◯ I prefer not to answer

What is the highest level of education that your parents have completed?

- ◯ Grade 8 or less
- ◯ Some high school
- ◯ High school graduate or GED
- ◯ Some post-high-school, no degree
- ◯ Associates degree
- ◯ Bachelors degree
- ◯ Professional school or graduate school
- ◯ Don't know

Are you currently employed?

- ◯ Employed for wages
- ◯ Self-employed
- ◯ Out of work and looking for work
- ◯ Out of work but not currently looking for work
- ◯ A homemaker
- ◯ Military
- ◯ Unable to work

Including wages, salaries, self-employment, and any other sources of income, what was your total income during the last 12 months?

- ◯ Less than $25,000
- ◯ $25,000-$65,000
- ◯ $65,000-$145,000
- ◯ $145,000-$225,000
- ◯ Over $225,000
- ◯ I prefer not to answer

What is your marital status?

- ◯ Single, never married
- ◯ Married
- ◯ In a domestic partnership
- ◯ Separated
- ◯ Divorced
- ◯ Widowed
- ◯ I prefer not to answer

How many people (including yourself) live in your residence?

- ◯ 1
- ◯ 2
- ◯ 3
- ◯ 4
- ◯ 5
- ◯ 6
- ◯ 7
- ◯ 8
- ◯ 9
- ◯ 10
- ◯ more than 10

Please confirm your age again. How old are you?

____

We are concerned that you may be under the age of 13 years. Unfortunately you are not eligible to take this survey and the survey will stop. Thank you for your time!

◯ Click here to close this page

In the following two questions please select the two social media and streaming platforms that you use the most on a weekly basis.

Which social media or streaming platform do you spend the most time on each week?

◯ Twitch
◯ Mixer
◯ Facebook
◯ Facebook Live
◯ YouTube
◯ YouTube Gaming
◯ Twitter
◯ Instagram
◯ Reddit
◯ Snapchat
◯ Vimeo
◯ Metacafe
◯ Smashcast tv
◯ Dailymotion
◯ InstaGib tv
◯ Steam Powered
◯ Origin
◯ Picarto

Which social media or streaming platform do you spend the second most time on per week?

◯ Twitch
◯ Mixer
◯ Facebook
◯ Facebook Live
◯ YouTube
◯ YouTube Gaming
◯ Twitter
◯ Instagram
◯ Reddit
◯ Snapchat
◯ Vimeo
◯ Metacafe
◯ Smashcast tv
◯ Dailymotion
◯ InstaGib tv
◯ Steam Powered
◯ Origin
◯ Picarto

Please go back and select two different social media platforms for the last two questions. This message will disappear when that is fixed.

Now please move onto the following questions and answer based upon what you selected as your most used streaming and social media platforms.

How many hours do you spend on Twitch daily?

- ◯ 0-2 hours
- ◯ 2-4 hours
- ◯ 4-6 hours
- ◯ 6-8 hours
- ◯ 8-10 hours
- ◯ 10-12 hours
- ◯ 12-18 hours
- ◯ 18-24 hours

On average how many hours do you spend on [social_media_platform_1] daily?

◯ 0-2 hours ◯ 2-4 hours
◯ 4-6 hours ◯ 6-8 hours
◯ 8-10 hours ◯ 10-12 hours
◯ 12-18 hours ◯ 18-24 hours

On average how many hours do you spend on [social_media_platform_2] daily?

- ◯ 0-2 hours
- ◯ 2-4 hours
- ◯ 4-6 hours
- ◯ 6-8 hours
- ◯ 8-10 hours
- ◯ 10-12 hours
- ◯ 12-18 hours
- ◯ 18-24 hours

Please select the answer C in this question

◯ A ◯ B ◯ C ◯ D ◯ E

Do you currently subscribe to any channels on Twitch?

◯ Yes
◯ No

How many Twitch channels do you subscribe to?
(Please enter a number)

◯ 0
◯ 1
◯ 2
◯ 3
◯ 4
◯ 5
◯ 6
◯ 7
◯ 8
◯ 9
◯ 10
◯ 11
◯ 12
◯ 13
◯ 14
◯ 15
◯ 16
◯ 17
◯ 18
◯ 19
◯ 20
◯ more than 20

Please go back enter the correct number of Twitch channels you subscribe to. If you don't subscribe to any channels please change your answer to the prior question. This message will disappear when that is fixed.

Is your Twitch account connected to an Amazon Prime account?

◯ Yes
◯ No

Is one of your Twitch subscriptions your free Amazon Prime subscription?

◯ Yes
◯ No

Please list 3 Twitch channels you watch the most:

____

____

____

Do you ever purchase bits on Twitch?

○ Yes
○ No
○ I don't know what bits are

About how many bits do you typically purchase in a year?

○ 100
○ 500
○ 1,500
○ 5,000
○ 10,000
○ 25,000
○ 25,000+

How often do you EAT (OR DRINK) the following items while watching Twitch?

Fast food	◯ Never<br>◯ Rarely<br>◯ Sometimes<br>◯ A lot
Snack foods like chips, cheese puffs, cookies	◯ Never<br>◯ Rarely<br>◯ Sometimes<br>◯ A lot
Candy	◯ Never<br>◯ Rarely<br>◯ Sometimes<br>◯ A lot
Self-prepared meals	◯ Never<br>◯ Rarely<br>◯ Sometimes<br>◯ A lot
Soda	◯ Never<br>◯ Rarely<br>◯ Sometimes<br>◯ A lot
Energy drinks	◯ Never<br>◯ Rarely<br>◯ Sometimes<br>◯ A lot
Coffee or Teas	◯ Never<br>◯ Rarely<br>◯ Sometimes<br>◯ A lot
Sports drinks	◯ Never<br>◯ Rarely<br>◯ Sometimes<br>◯ A lot
Other drinks	◯ Never<br>◯ Rarely<br>◯ Sometimes<br>◯ A lot

How often do you order food from meal delivery services (i.e., GrubHub, DoorDash, etc.) while watching Twitch?

- ◯ Never
- ◯ Rarely
- ◯ Sometimes
- ◯ Often

How often do you SEE ADVERTISING FOR the following items while watching Twitch?

Fast food	◯ Never<br>◯ Rarely<br>◯ Sometimes<br>◯ Often
Food delivery services	◯ Never<br>◯ Rarely<br>◯ Sometimes<br>◯ Often
Snack foods	◯ Never<br>◯ Rarely<br>◯ Sometimes<br>◯ Often
Candy	◯ Never<br>◯ Rarely<br>◯ Sometimes<br>◯ Often
Soda	◯ Never<br>◯ Rarely<br>◯ Sometimes<br>◯ Often
Energy Drinks	◯ Never<br>◯ Rarely<br>◯ Sometimes<br>◯ Often
Coffees, or Teas	◯ Never<br>◯ Rarely<br>◯ Sometimes<br>◯ Often
Sports drinks	◯ Never<br>◯ Rarely<br>◯ Sometimes<br>◯ Often
Other drinks	◯ Never<br>◯ Rarely<br>◯ Sometimes<br>◯ Often

Which of the following brands have you seen advertised on Twitch? Check all that apply:

- ☐ Burger King
- ☐ Chex
- ☐ Chick-fil-A
- ☐ Chipotle
- ☐ Coca-Cola
- ☐ Dr. Pepper
- ☐ Fanta
- ☐ GamerFuel (Mountain Dew)
- ☐ Gfuel
- ☐ GrubHub
- ☐ Hershey's
- ☐ KFC
- ☐ Kit Kat
- ☐ Kool-Aid
- ☐ Madrinas Coffee
- ☐ McDonalds
- ☐ Monster Energy
- ☐ Nutella
- ☐ Oreo
- ☐ Pepsi
- ☐ Pringles
- ☐ RedBull
- ☐ Reese's
- ☐ Skittles
- ☐ TacoBell
- ☐ Totino's
- ☐ Twix
- ☐ UberEats
- ☐ Wendy's
- ☐ none of the above

List here any other brands you have seen advertised on Twitch

---

After seeing advertisements on Twitch do you crave any of the products that you see?

- ◯ Yes
- ◯ No

If yes, please indicate which ones

- ☐ Burger King
- ☐ Chex
- ☐ Chick-fil-A
- ☐ Chipotle
- ☐ Coca-Cola
- ☐ Dr. Pepper
- ☐ Fanta
- ☐ GamerFuel (Mountain Dew)
- ☐ Gfuel
- ☐ GrubHub
- ☐ Hershey's
- ☐ KFC
- ☐ Kit Kat
- ☐ Kool-Aid
- ☐ Madrinas Coffee
- ☐ McDonalds
- ☐ Monster Energy
- ☐ Nutella
- ☐ Oreo
- ☐ Pepsi
- ☐ Pringles
- ☐ RedBull
- ☐ Reese's
- ☐ Skittles
- ☐ TacoBell
- ☐ Totino's
- ☐ Twix
- ☐ UberEats
- ☐ Wendy's
- ☐ Other

If other, please list any other products here

Do you ever purchase products because you have seen them advertised on Twitch?

- ◯ Yes
- ◯ No

If yes, please indicate which ones

- ☐ Burger King
- ☐ Chex
- ☐ Chick-fil-A
- ☐ Chipotle
- ☐ Coca-Cola
- ☐ Dr. Pepper
- ☐ Fanta
- ☐ GamerFuel (Mountain Dew)
- ☐ Gfuel
- ☐ GrubHub
- ☐ Hershey's
- ☐ KFC
- ☐ Kit Kat
- ☐ Kool-Aid
- ☐ Madrinas Coffee
- ☐ McDonalds
- ☐ Monster Energy
- ☐ Nutella
- ☐ Oreo
- ☐ Pepsi
- ☐ Pringles
- ☐ RedBull
- ☐ Reese's
- ☐ Skittles
- ☐ TacoBell
- ☐ Totino's
- ☐ Twix
- ☐ UberEats
- ☐ Wendy's
- ☐ Other

If other, please list any other products here

How often do you use chat/comments while watching Twitch?	◯ Never<br>◯ Rarely<br>◯ Sometimes<br>◯ Often
How often do you see other users talking about specific foods/beverage products in chat/comments while watching Twitch?	◯ Never<br>◯ Rarely<br>◯ Sometimes<br>◯ Often
How often do you talk about specific foods/beverage products in chat/comments while watching Twitch?	◯ Never<br>◯ Rarely<br>◯ Sometimes<br>◯ Often

Do you participate in mini-games or promotional events for products on Twitch?

◯ Yes
◯ No

Fill in the blank: "On Twitch there is ______ advertising."

◯ Too little
◯ Just enough
◯ Too much

Fill in the blank: "When I see advertising on Twitch, I am _______."

◯ Happy
◯ Annoyed
◯ Doesn't bother me

Please rate your agreement with these 3 statements.

The main purpose of advertising on Twitch is to:

	Strongly disagree	Disagree	Agree	Strongly agree
promote products	○	○	○	○
support streamers/content creators	○	○	○	○
increase profits for the website or app	○	○	○	○

Great Job -- You're half way done. Keep going, and we can get you entered to win an Amazon gift card!

These next items are about [social_media_platform_1]

How often do you EAT (OR DRINK) the following items while watching [social_media_platform_1]?

Fast food	◯ Never<br>◯ Rarely<br>◯ Sometimes<br>◯ A lot
Snack foods like chips, cheese puffs, cookies	◯ Never<br>◯ Rarely<br>◯ Sometimes<br>◯ A lot
Candy	◯ Never<br>◯ Rarely<br>◯ Sometimes<br>◯ A lot
Self-prepared meals	◯ Never<br>◯ Rarely<br>◯ Sometimes<br>◯ A lot
Soda	◯ Never<br>◯ Rarely<br>◯ Sometimes<br>◯ A lot
Energy drinks	◯ Never<br>◯ Rarely<br>◯ Sometimes<br>◯ A lot
Coffee and Teas	◯ Never<br>◯ Rarely<br>◯ Sometimes<br>◯ A lot
Sports drinks	◯ Never<br>◯ Rarely<br>◯ Sometimes<br>◯ A lot
Other drinks	◯ Never<br>◯ Rarely<br>◯ Sometimes<br>◯ A lot

How often do you order food from meal delivery services (i.e., GrubHub, DoorDash, etc.) while watching [social_media_platform_1]?	◯ Never<br>◯ Rarely<br>◯ Sometimes<br>◯ Often

How often do you SEE ADVERTISING FOR the following items while watching [social_media_platform_1]?

Fast food	◯ Never<br>◯ Rarely<br>◯ Sometimes<br>◯ Often
Food delivery services	◯ Never<br>◯ Rarely<br>◯ Sometimes<br>◯ Often
Snack foods	◯ Never<br>◯ Rarely<br>◯ Sometimes<br>◯ Often
Candy	◯ Never<br>◯ Rarely<br>◯ Sometimes<br>◯ Often
Soda	◯ Never<br>◯ Rarely<br>◯ Sometimes<br>◯ Often
Energy Drink	◯ Never<br>◯ Rarely<br>◯ Sometimes<br>◯ Often
Coffees, or Teas	◯ Never<br>◯ Rarely<br>◯ Sometimes<br>◯ Often
Sports drinks	◯ Never<br>◯ Rarely<br>◯ Sometimes<br>◯ Often
Other drinks	◯ Never<br>◯ Rarely<br>◯ Sometimes<br>◯ Often

Which of the following brands have you seen advertised on [social_media_platform_1]? Check all that apply:

☐ Burger King
☐ Chex
☐ Chick-fil-A
☐ Chipotle
☐ Coca-Cola
☐ Dr. Pepper
☐ Fanta
☐ GamerFuel (Mountain Dew)
☐ Gfuel
☐ GrubHub
☐ Hershey's
☐ KFC
☐ Kit Kat
☐ Kool-Aid
☐ Madrinas Coffee
☐ McDonalds
☐ Monster Energy
☐ Nutella
☐ Oreo
☐ Pepsi
☐ Pringles
☐ RedBull
☐ Reese's
☐ Skittles
☐ TacoBell
☐ Totino's
☐ Twix
☐ UberEats
☐ Wendy's
☐ none of the above

List here any other brands you have seen advertised on [social_media_platform_1]

After seeing advertisements on [social_media_platform_1] do you crave any of the products that you see?

◯ Yes
◯ No

If yes, please indicate which ones

- ☐ Burger King
- ☐ Chex
- ☐ Chick-fil-A
- ☐ Chipotle
- ☐ Coca-Cola
- ☐ Dr. Pepper
- ☐ Fanta
- ☐ GamerFuel (Mountain Dew)
- ☐ Gfuel
- ☐ GrubHub
- ☐ Hershey's
- ☐ KFC
- ☐ Kit Kat
- ☐ Kool-Aid
- ☐ Madrinas Coffee
- ☐ McDonalds
- ☐ Monster Energy
- ☐ Nutella
- ☐ Oreo
- ☐ Pepsi
- ☐ Pringles
- ☐ RedBull
- ☐ Reese's
- ☐ Skittles
- ☐ TacoBell
- ☐ Totino's
- ☐ Twix
- ☐ UberEats
- ☐ Wendy's
- ☐ Other

If other, please list any other products here

Do you ever purchase products because you have seen them advertised on [social_media_platform_1]?

- ◯ Yes
- ◯ No

If yes, please indicate which ones

- ☐ Burger King
- ☐ Chex
- ☐ Chick-fil-A
- ☐ Chipotle
- ☐ Coca-Cola
- ☐ Dr. Pepper
- ☐ Fanta
- ☐ GamerFuel (Mountain Dew)
- ☐ Gfuel
- ☐ GrubHub
- ☐ Hershey's
- ☐ KFC
- ☐ Kit Kat
- ☐ Kool-Aid
- ☐ Madrinas Coffee
- ☐ McDonalds
- ☐ Monster Energy
- ☐ Nutella
- ☐ Oreo
- ☐ Pepsi
- ☐ Pringles
- ☐ RedBull
- ☐ Reese's
- ☐ Skittles
- ☐ TacoBell
- ☐ Totino's
- ☐ Twix
- ☐ UberEats
- ☐ Wendy's
- ☐ Other

If other, please list any other products here

How often do you use chat/comments while watching [social_media_platform_1]?

◯ Never
◯ Rarely
◯ Sometimes
◯ Often

How often do you see other users talking about specific foods/beverage products in chat/comments while watching [social_media_platform_1]?

◯ Never
◯ Rarely
◯ Sometimes
◯ Often

How often do you talk about specific foods/beverage products in chat/comments while watching [social_media_platform_1]?

◯ Never
◯ Rarely
◯ Sometimes
◯ Often

Do you participate in mini-games or promotional events for products on [social_media_platform_1]?

◯ Yes
◯ No

Fill in the blank: "On [social_media_platform_1] there is ______ advertising."

◯ Too little
◯ Just enough
◯ Too much

Fill in the blank: "When I see advertising on [social_media_platform_1], I am _______."

◯ Happy
◯ Annoyed
◯ Doesn't bother me

Please rate your agreement with these 3 statements.

The main purpose of advertising on [social_media_platform_1] is to:

	Strongly disagree	Disagree	Agree	Strongly agree
promote products	○	○	○	○
support streamers/content creators	○	○	○	○
increase profits for the website or app	○	○	○	○

You've made it 75% of the way through, only 40 more questions left! You can do this!

These next items are about [social_media_platform_2]

How often do you EAT (OR DRINK) the following items while watching [social_media_platform_2]?

Fast food	◯ Never<br>◯ Rarely<br>◯ Sometimes<br>◯ A lot
Snack foods like chips, cheese puffs, cookies	◯ Never<br>◯ Rarely<br>◯ Sometimes<br>◯ A lot
Candy	◯ Never<br>◯ Rarely<br>◯ Sometimes<br>◯ A lot
Self-prepared meals	◯ Never<br>◯ Rarely<br>◯ Sometimes<br>◯ A lot
Soda	◯ Never<br>◯ Rarely<br>◯ Sometimes<br>◯ A lot
Energy drinks	◯ Never<br>◯ Rarely<br>◯ Sometimes<br>◯ A lot
Coffee and Teas	◯ Never<br>◯ Rarely<br>◯ Sometimes<br>◯ A lot
Sports drinks	◯ Never<br>◯ Rarely<br>◯ Sometimes<br>◯ A lot
Other drinks	◯ Never<br>◯ Rarely<br>◯ Sometimes<br>◯ A lot
How often do you order food from meal delivery services (i.e., GrubHub, DoorDash, etc.) while watching [social_media_platform_2]?	◯ Never<br>◯ Rarely<br>◯ Sometimes<br>◯ Often

How often do you SEE ADVERTISING FOR the following items while watching [social_media_platform_2]?

Fast food	◯ Never<br>◯ Rarely<br>◯ Sometimes<br>◯ Often
Food delivery services	◯ Never<br>◯ Rarely<br>◯ Sometimes<br>◯ Often
Snack foods	◯ Never<br>◯ Rarely<br>◯ Sometimes<br>◯ Often
Candy	◯ Never<br>◯ Rarely<br>◯ Sometimes<br>◯ Often
Soda	◯ Never<br>◯ Rarely<br>◯ Sometimes<br>◯ Often
Energy Drinks	◯ Never<br>◯ Rarely<br>◯ Sometimes<br>◯ Often
Coffees, or Teas	◯ Never<br>◯ Rarely<br>◯ Sometimes<br>◯ Often
Sports drinks	◯ Never<br>◯ Rarely<br>◯ Sometimes<br>◯ Often
Other drinks	◯ Never<br>◯ Rarely<br>◯ Sometimes<br>◯ Often

Which of the following brands have you seen advertised on [social_media_platform_2]? Check all that apply:

☐ Burger King
☐ Chex
☐ Chick-fil-A
☐ Chipotle
☐ Coca-Cola
☐ Dr. Pepper
☐ Fanta
☐ GamerFuel (Mountain Dew)
☐ Gfuel
☐ GrubHub
☐ Hershey's
☐ KFC
☐ Kit Kat
☐ Kool-Aid
☐ Madrinas Coffee
☐ McDonalds
☐ Monster Energy
☐ Nutella
☐ Oreo
☐ Pepsi
☐ Pringles
☐ RedBull
☐ Reese's
☐ Skittles
☐ TacoBell
☐ Totino's
☐ Twix
☐ UberEats
☐ Wendy's
☐ none of the above

List here any other brands you have seen advertised on [social_media_platform_2]

After seeing advertisements on [social_media_platform_2] do you crave any of the products that you see?

◯ Yes
◯ No

If yes, please indicate which ones

☐ Burger King
☐ Chex
☐ Chick-fil-A
☐ Chipotle
☐ Coca-Cola
☐ Dr. Pepper
☐ Fanta
☐ GamerFuel (Mountain Dew)
☐ Gfuel
☐ GrubHub
☐ Hershey's
☐ KFC
☐ Kit Kat
☐ Kool-Aid
☐ Madrinas Coffee
☐ McDonalds
☐ Monster Energy
☐ Nutella
☐ Oreo
☐ Pepsi
☐ Pringles
☐ RedBull
☐ Reese's
☐ Skittles
☐ TacoBell
☐ Totino's
☐ Twix
☐ UberEats
☐ Wendy's
☐ Other

If other, please list any other products here

Do you ever purchase products because you have seen them advertised on [social_media_platform_2]?

○ Yes
○ No

If yes, please indicate which ones

- [ ] Burger King
- [ ] Chex
- [ ] Chick-fil-A
- [ ] Chipotle
- [ ] Coca-Cola
- [ ] Dr. Pepper
- [ ] Fanta
- [ ] GamerFuel (Mountain Dew)
- [ ] Gfuel
- [ ] GrubHub
- [ ] Hershey's
- [ ] KFC
- [ ] Kit Kat
- [ ] Kool-Aid
- [ ] Madrinas Coffee
- [ ] McDonalds
- [ ] Monster Energy
- [ ] Nutella
- [ ] Oreo
- [ ] Pepsi
- [ ] Pringles
- [ ] RedBull
- [ ] Reese's
- [ ] Skittles
- [ ] TacoBell
- [ ] Totino's
- [ ] Twix
- [ ] UberEats
- [ ] Wendy's
- [ ] Other

If other, please list any other products here

How often do you use chat/comments while watching [social_media_platform_2]?	◯ Never<br>◯ Rarely<br>◯ Sometimes<br>◯ Often
How often do you see other users talking about specific foods/beverage products in chat/comments while watching [social_media_platform_2]?	◯ Never<br>◯ Rarely<br>◯ Sometimes<br>◯ Often
How often do you talk about specific foods/beverage products in chat/comments while watching [social_media_platform_2]?	◯ Never<br>◯ Rarely<br>◯ Sometimes<br>◯ Often

Do you participate in mini-games or promotional events for products on [social_media_platform_2]?

◯ Yes
◯ No

Fill in the blank: "On [social_media_platform_2] there is ______ advertising."

◯ Too little
◯ Just enough
◯ Too much

Fill in the blank: "When I see advertising on [social_media_platform_2], I am _______."

◯ Happy
◯ Annoyed
◯ Doesn't bother me

Please rate your agreement with these 3 statements.

The main purpose of advertising on [social_media_platform_2] is to:

	Strongly disagree	Disagree	Agree	Strongly agree
promote products	○	○	○	○
support streamers/content creators	○	○	○	○
increase profits for the website or app	○	○	○	○

Thank you for completing the Twitch and social media section of the survey!

Please continue and answer the following questions about food and eating habits.

I deliberately take small helpings as a means of controlling my weight.	◯ Definitely true<br>◯ Mostly true<br>◯ Mostly false<br>◯ Definitely false
I consciously hold back at meals in order not to gain weight.	◯ Definitely true<br>◯ Mostly true<br>◯ Mostly false<br>◯ Definitely false
I do not eat some foods because they make me fat.	◯ Definitely true<br>◯ Mostly true<br>◯ Mostly false<br>◯ Definitely false
How frequently do you avoid 'stocking up' on tempting foods?	◯ Almost never<br>◯ Rarely<br>◯ Usually<br>◯ Almost always
How likely are you to consciously eat less than you want?	◯ Unlikely<br>◯ Slightly likely<br>◯ Moderately likely<br>◯ Very likely

On a scale of 1 to 8, where 1 means no restraint in eating (eating whatever you want, whenever you want it) and 8 means total restraint (constantly limiting food intake and never 'giving in'), what number would you give yourself?	◯ 1- Eat whatever I want, whenever I want to<br>◯ 2<br>◯ 3<br>◯ 4<br>◯ 5<br>◯ 6<br>◯ 7<br>◯ 8- Constantly limiting food intake, never 'giving in'
When I smell a sizzling steak or a juicy piece of meat, I find it very difficult to keep from eating, even if I have just finished a meal.	◯ Definitely true<br>◯ Mostly true<br>◯ Mostly false<br>◯ Definitely false
Sometimes when I start eating, I just can't seem to stop	◯ Definitely true<br>◯ Mostly true<br>◯ Mostly false<br>◯ Definitely false
Being with someone who is eating often makes me hungry enough to eat also.	◯ Definitely true<br>◯ Mostly true<br>◯ Mostly false<br>◯ Definitely false
When I see a real delicacy, I often get so hungry that I have to eat right away.	◯ Definitely true<br>◯ Mostly true<br>◯ Mostly false<br>◯ Definitely false
I get so hungry that my stomach often seems like a bottomless pit.	◯ Definitely true<br>◯ Mostly true<br>◯ Mostly false<br>◯ Definitely false
I am always hungry so it is hard for me to stop eating before I finish the food on my plate.	◯ Definitely true<br>◯ Mostly true<br>◯ Mostly false<br>◯ Definitely false
I am always hungry enough to eat at any time.	◯ Definitely true<br>◯ Mostly true<br>◯ Mostly false<br>◯ Definitely false
How often do you feel hungry?	◯ Never<br>◯ Rarely<br>◯ Sometimes<br>◯ Often
Do you go on eating binges though you are not hungry?	◯ Never<br>◯ Rarely<br>◯ Sometimes<br>◯ Often

When I feel anxious, I find myself eating.	◯ Definitely true<br>◯ Mostly true<br>◯ Mostly false<br>◯ Definitely false
When I feel blue, I often overeat.	◯ Definitely true<br>◯ Mostly true<br>◯ Mostly false<br>◯ Definitely false
When I feel lonely, I console myself by eating.	◯ Definitely true<br>◯ Mostly true<br>◯ Mostly false<br>◯ Definitely false
Please select the answer A in this question	◯ A<br>◯ B<br>◯ C<br>◯ D<br>◯ E

Only 20 questions left, you're almost done!

Please respond to the following statements based upon if it is rarely, sometimes, often, or a lot of the time true for you regarding food and eating habits.

	Rarely	Sometimes	Often	A lot
I want foods or drinks that I see others eating	○	○	○	○
I notice snack or drink vending machines	○	○	○	○
I want to eat when people talk about food	○	○	○	○
I notice restaurant signs/logos	○	○	○	○
I notice snacks at check-out aisles	○	○	○	○
I like certain snacks because of the packaging	○	○	○	○
I notice the sound of food cooking	○	○	○	○
I want to eat when I hear a snack being opened	○	○	○	○
I expect to snack when I'm in my car	○	○	○	○

Please answer the following statements based on whether they are never, rarely, sometimes, often, or very often true for you.

	Never	Rarely	Sometimes	Often
If food tastes good to you, do you eat more than usual?	○	○	○	○
If food smells and looks good, do you eat more than usual?	○	○	○	○
If you see or smell something delicious, do you have a desire to eat it?	○	○	○	○
If you have something delicious to eat, do you eat it straight away?	○	○	○	○
If you walk past the baker do you have the desire to buy something delicious?	○	○	○	○

Last 5 questions!

Please answer the following statements based on whether they are never, rarely, sometimes, often, or very often true for you.

	Never	Rarely	Sometimes	Often
If you walk past a snackbar or a café, do you have the desire to buy something delicious?	○	○	○	○
If you see others eating, do you also have the desire to eat?	○	○	○	○
Can you resist eating delicious foods?	○	○	○	○
Do you eat more than usual, when you see others eating?	○	○	○	○
When preparing a meal are you inclined to eat something?	○	○	○	○

That's it!

Thank you for your participation in this online research survey!

If you would like to be entered into the drawing for one of the ten $50 Amazon gift cards, please enter your email here. If you don't want to enter the raffle, just close this page.

Email address:

____

Please re-enter your email address:

____
