# Supplementary material for: Twitch user perceptions, attitudes and behaviours in relation to food and beverage marketing on Twitch compared with YouTube
Source: J Nutr Sci. 2021 Apr 27;10:e32. doi: 10.1017/jns.2021.22 (PMC8141682; doi:10.1017/jns.2021.22)
Supplement: Supplementary file 1 [file jnssup.zip › S2048679021000227sup001.docx]

**Appendix A. Blank Version of the Survey**

See the attached document for a blank version of the survey provided to users

**Appendix B. Sub-Analysis by Binary Pay Tier with Collapsed and Extended Likert Scale**

Note: “Casual” users denote users who do not pay for the platform, while “Active” users denote individuals who do pay for the platform.

**Appendix C. Sub-Analysis with Multi-Category Pay Tier with Collapsed and Extended Likert Scale**

Note: “Background” users are those who do not pay for the platform through subscriptions or Bits, “Casual” users are those who do not pay for Bits and only have one free subscription through their Amazon Prime account, and “Active” users are those that pay for the platform.

**Appendix D.** Viewership Hours with Extended Likert Scale
